# Relation between Charging Times and Storage Properties of Nanoporous Supercapacitors

**DOI:** 10.3390/nano12040587

**Published:** 2022-02-09

**Authors:** Timur Aslyamov, Konstantin Sinkov, Iskander Akhatov

**Affiliations:** 1 Skolkovo Institute of Science and Technology, Bolshoy Boulevard 30, Bld. 1, 121205 Moscow, Russia; I.Akhatov@skoltech.ru; 2 Schlumberger Moscow Research, Leningradskoe Shosse 16A/3, 125171 Moscow, Russia; sinkovk@gmail.com

**Keywords:** supercapacitors, nanoporous electrodes, charging time

## Abstract

An optimal combination of power and energy characteristics is beneficial for the further progress of supercapacitors-based technologies. We develop a nanoscale dynamic electrolyte model, which describes both static capacitance and the time-dependent charging process, including the initial square-root dependency and two subsequent exponential trends. The observed charging time corresponds to one of the relaxation times of the exponential regimes and significantly depends on the pore size. Additionally, we find analytical expressions providing relations of the time scales to the electrode’s parameters, applied potential, and the final state of the confined electrolyte. Our numerical results for the charging regimes agree with published computer simulations, and estimations of the charging times coincide with the experimental values.

## 1. Introduction

Among all modern energy sources, supercapacitors demonstrate an extraordinary power density and an extremely long cycling life [1]. Such rapid charging and discharging occur due to fast adsorption-electrostatic processes, making supercapacitor technology environmentally friendly. These advantages open up a wide range of possible applications from small devices [2] to electric cars [3]. However, the widespread use of supercapacitor technology is limited by their relatively low energy density [4]. The implementation of nanoporous electrodes led to a severe increase in energy density through a significant increase in capacitance, first experimentally observed in sub-nanoporous carbon materials [5]. Moreover, subsequent experiments showed that the pore size giving the highest capacitance corresponds to the diameter of the electrolyte molecules [6]. The capacitance’s oscillatory behavior as a function of pore size has been successfully described in terms of the classical density functional theory (c-DFT) [7], accounting for confined properties of the charged hard spheres at an applied electrostatic potential. The state of the art c-DFT approach [8] allows one to investigate how the supercapacitor parameters, namely electrodes’ pore sizes [9,10] and electrolyte composition [11,12,13], affect energy storage performance.

In spite of the long history of [14,15,16] studies of porous electrode charging, the vast majority of existing dynamic models correspond to meso- and macropores (pore size H≥2nm), where the properties of confined dilute electrolytes are similar to the bulk ones. In this case, the charging time is often estimated using the transmission line model (TLM), first proposed and thoroughly studied in Refs. [14,15,16]. The TLM treats the electrolyte-filled pore as an equivalent circuit of resistors and capacitors. The corresponding total resistance *R* and capacitance *C* take into account the properties of the electrolyte in the bulk and double electric layer (EDL) states, respectively. More than half a century ago, de Levy proposed a diffusion equation for the electrostatic potential difference between the pore surface and the central plane, which leads to a time scale RC [14,15]. Significantly, the effective circuit is used nowadays to investigate the various properties of the electrolyte charging [17,18,19]. Biesheuvel and Bazant extended transmission line approach considering pure capacitive process Ref. [20] and Faradaic reaction Refs. [21,22] in porous electrodes. For low applied potentials U≪25 mV model [20] results in the TLM, which is the linearization of Poisson–Nernst–Planck (PNP) equations [23] at this limit. Because of the assumption of low applied potential, TLM misses some critical phenomena [24] at conditions corresponding to the majority of applications U∼0.1–1 V. For example, the nonlinear model [20] predicts the slower charging at higher potentials, which is crucial for the power performance. However, the PNP calculations [24] showed that this predicted charging slowdown is overestimated due to the unaccounted surface conductivity. Thus, an accurate description of the electrolyte–electrode interface is crucial for adequately modeling nanopore charging at moderate/high applied potentials.

Very recently, the TLM approach has been applied to nanoporous electrodes (*H* ∼ 2 nm), and realistic scaling of the charging time has been obtained in terms of physically determined parameters [19]. The authors of ref. [19] have also shown that two comparable timescales exist at high applied potentials: the first one corresponds to the equivalent circuit model, and the second timescale is related to adsorption. A more detailed analysis of the charge dynamics in nanopores requires information on the packing properties of finite-sized ions, which goes beyond the [19] TLM models. The most realistic description of ion transport in nanopores was obtained using the molecular dynamics (MD) method. Molecular dynamics (MD) simulations [25,26] demonstrated that the charging of the electrolyte filled pores is described by three consecutive regimes (modes). More precisely, the charging starts with ions diffusion characterized by the slow squared-root dependence of the total charge on time. Further, ion transport corresponds to two exponential regimes at the intermediate and late times. The second exponential regime with a higher relaxation time becomes crucial in ultra-narrow pores, which results in a significant slowdown of the charging. The authors of ref. [27] demonstrated how to speed up the nanopores charging using time-dependent applied potential. Such control of the applied potential allows the ions to avoid the molecular clogging which causes the sluggish dynamics. Therefore, modeling the ions’ transport inside nanopores is critical to designing the supercapacitors with the optimal relation between storage and power properties.

In addition, an actual application of the ion transport models is capacitive deionization technologies which use an electrostatic field to remove salt ions and provide the water desalination during the filtration through porous electrodes [28]. Additionally, the recent experiments [29] demonstrated that the salt ions could be excluded from the confined water because of the steric effects of the nano-scale slit pores. Thus, it is crucial to combine the electrostatic and steric properties to model the ion transport inside the nanopores properly.

This paper describes the ion dynamics inside a nanopore as wide as a molecule. Such sub-nanoporous electrodes are necessary for further advances in supercapacitor-based technologies. However, the sub-nanopore size limitation causes a significant slowing down of ion motion and, consequently, the charge. We develop a theory based on the time-dependent version of the c-DFT approach (see refs. [30,31] for one of the first derivations, and ref. [32] for the comprehensive literature review). More precisely, we derive and numerically solve the transport equations for the charged ions in the three-dimensional narrow slit pores. Such pores exhibit negligibly small values of the width-to-length ratio, which allows us to derive an asymptotic model—a one-dimensional transport equation reflecting the properties of the ion dynamics inside 3D slit nanopores. The proposed theory describes three consecutive charging regimes: an initial root-square process and two exponential regimes with different time scales. The last exponential regime plays a crucial role in the ultra-narrow pores (pore width is around ion diameter) and results in the charging slowdown. Before the current work, the details of the charging inside ultra-narrow pores had been investigated using MD simulations only [25,26,27]. Indeed, since we use an accurate thermodynamic potential, our model accounts for the electric and packaging properties of finite-sized ions within the nanopores. It is an advantage over recently published dynamic models [18,19], and allows us to relate charging times to the given characteristics of nanopore supercapacitors.

## 2. Model

**Figure 1:**
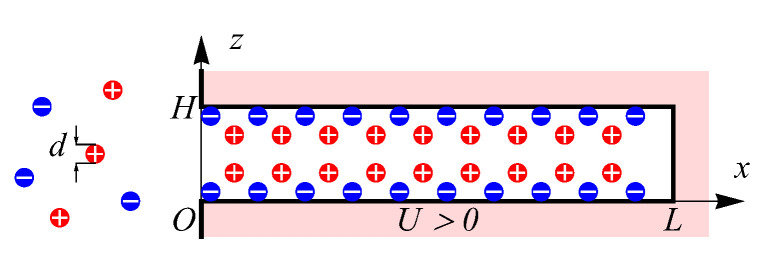
Slit pore connected to bulk volume of symmetric electrolyte, constant electric potential is applied to the pore walls.

We use the slit pore geometry shown in Figure 1, which describes the spatial structure of modern porous materials, such as graphene based electrodes [33,34] and graphene oxide (GO)/MXene fibers [35,36], exhibiting aligned slit-nanopores of width *H* comparable to electrolyte diameter *d* and length L≫H. The considered materials are used in supercapacitor’s technologies as the porous electrodes which accumulate charge through electric double layer formation without chemical reactions. More precisely, these materials show promising applications for wearable electronics and smart textiles devices due to outstanding energy density and high flexibility [37]. Moreover, our geometrical representation of the pore closely resembles recent molecular dynamic simulations of the supercapacitors [26,27], which allows us to make a comparison of our predictions with the MD-simulated electrolyte’s behavior. The electrodes with inhomogeneous pore-network considered in refs. [21,38,39] are beyond the scope of our work. However, the developed model of individual slit nanopore charging can be implemented as a pore-network building block in the case of a non-trivial pore-size distribution [39]. A possible implementation follows from ref. [39], where our transport equations can replace the Navier–Stokes equation for the effective medium approximation—the porous reservoir is a uniform network of equal short pores.

In the case of slit pore geometry (Figure 1), the dynamic component density $\rho_{i}\left(t,x,z\right)$ for i=1,⋯,n (where *n* is the number of components) depends on the coordinate *x* in lateral direction along the pore’s surface and the normal distance to the surface *z*. Notice, that we assume the homogeneous distribution in the direction which is perpendicular to the *x*- and *z*-coordinates, see Figure 1. Therefore, to describe dynamics of the charging process, we apply the time-dependent version of c-DFT [40] in two spatial coordinates (x,z), defined in the general form as

(1) $$\partial_{t}\rho_{i}-\beta D_{i}\nabla \left(\rho_{i}\nabla \mu_{i}\right)=0,$$

where $D_{i}$ is the diffusion coefficient of *i*-th component, $\beta =1/k_{B}T$, $k_{B}$ is the Boltzmann constant, *T* is the temperature, $\nabla =\{\partial_{x},\partial_{z}\}$ is 2D gradient vector, and $\mu_{i}\left(t,x,z\right)$ is the local chemical potential of the *i*-th component.

To describe the confined electrolyte we use one of the most popular c-DFT approaches based on the confined hard sphere model [41] and an electrostatic extension [42] accounting for the contributions from Coulomb interaction and additional finite size residual correlations. The electrode’s pores are considered as an open system connected with a bulk electrolyte. In accordance with Ref. [40], the chemical potentials $\mu_{i}\left(t,x,z\right)$ have the following form

(2) $$\mu_{i}=k_{B}T\log\left(\rho_{i}\Lambda^{3}\right)+eZ_{i}\psi +\frac{\delta f_{\mathrm{exc}}}{\delta \rho_{i}},$$

where ψ is the electric potential, *e* is the proton charge, $Z_{i}$ is the ion valency, Λ is the de Broglie wavelength. The electric potential ψ(t,x,z) satisfies the Poisson equation

(3) $$\beta e\Delta \psi =-4\pi l_{B}\sum_{i=1}^{n}Z_{i}\rho_{i},$$

where $\Delta =\partial_{xx}+\partial_{zz}$ is the 2D Laplace operator and $l_{B}=\beta e^{2}/4\pi \epsilon \epsilon_{0}$ is the Bjerrum length. A detailed description of the used c-DFT approach including particular form of the excess term $f_{\mathrm{exc}}$ can be found in Appendix D. Additionally, see in Appendix D the static the capacitance’s properties from implemented c-DFT which correspond to model [9] and show the realistic behavior.

Initially, no electrostatic potentials are applied, and the total charge of the symmetric electrolyte is zero. We consider a step charge when the potential changes abruptly from zero to some positive value U>0. The applied potential causes the influx into the pores of oppositely charged ions called counter-ions (anions in the case of U>0), while ions with a charge of the same sign as the applied potential (co-ions, cations for U>0) are pushed out of the pore volume. Inside sub-nanopores, the local increase in the counter-ion density can strongly influence the co-ion desorption, hindering their release due to counter-ion clogging and thus leading to a notable slowdown of the charging process [26]. The main variables of interest are the charges associated with individual electrolyte components $Q_{i}\left(t\right)=-eZ_{i}L^{-1}\int_{0}^{L}\int_{0}^{H}dx\;dz\;\rho_{i}\left(t,x,z\right)$ and the surface total charge density $Q\left(t\right)=\sum_{i=1}^{n}Q_{i}\left(t\right)$. The final charge $Q_{\infty }$ corresponds to the steady distributions $\rho_{i}^{\infty }\left(t,x,z\right)\equiv \rho_{i}^{\infty }\left(z\right)$, which are in equilibrium with the bulk electrolyte and depend on the applied potential *U*.

Considering Equations (1) and (3) in the slit-pore geometry (see (A5) and (A6)) with very small ratio H/L≪1, we seek the solutions of Equation (1) in the following forms:

(4) $$\begin{array}{ccc} \rho_{i}\left(t,x,z\right) & = & \rho_{i}^{0}\left(t,x,z\right)+\frac{H^{2}}{L^{2}}\rho_{i}^{1}\left(t,x,z\right)+\cdots , \end{array}$$

(5) $$\begin{array}{ccc} \psi (t,x,z) & = & \psi^{0}\left(t,x,z\right)+\frac{H^{2}}{L^{2}}\psi^{1}\left(t,x,z\right)+\cdots . \end{array}$$

where upper indices correspond to the O(1) and $O(H^{2}/L^{2})$ problems. As one can see in Appendix A, we use the O(1) equations to find that $\mu_{i}^{0}\left(t,x,z\right)\equiv \mu_{i}^{0}\left(t,x\right)$ do not depend on the *z*-coordinate; and electrostatic potential $\psi^{0}\left(t,x,z\right)$ can be found from the O(1)-Poisson equation. Therefore, the O(1)-problem allows us to describe the *z*-coordinate dependence, while $O(H^{2}/L^{2})$ provides information about dynamics and *x*-coordinate distribution. One of our most striking results is the demonstration that a complete solution to the problem $O(H^{2}/L^{2})$ is not required. Indeed, we derive an asymptotic model describing charging dynamics in terms of the pore cross-section averaged quantities for the O(1)-variables $\rho_{i}^{0}\left(t,x,z\right)$ and $\mu_{i}^{0}\left(t,x,z\right)$, as follows (see details in Appendix A):

(6) $$\partial_{t}{\bar{\rho }}_{i}^{0}\left(t,x\right)-\beta D_{i}\partial_{x}\left({\bar{\rho }}_{i}^{0}\left(t,x\right)\partial_{x}\mu_{i}^{0}\left({\bar{\rho }}_{1}^{0},\cdots ,{\bar{\rho }}_{n}^{0}\right)\right)=0.$$

where, ${\bar{\rho }}_{i}^{0}\left(t,x\right)=\int_{0}^{H}dz\;\rho_{i}^{0}\left(t,x,z\right)/H$ are pore cross-section-averaged densities, and $\mu_{i}^{0}={\bar{\mu }}_{i}\left({\bar{\rho }}_{1},...,{\bar{\rho }}_{n}\right)$ are functions of averaged densities only, see Appendix B. We close the 1D transport equation by the following initial and boundary conditions:

(7) $$\begin{array}{ccc} {\bar{\rho }}_{i}^{0}\left(t,0\right) & = & {\bar{\rho }}_{i}^{\infty }\left(U\right), \end{array}$$

(8) $$\begin{array}{ccc} \partial_{x}{\bar{\mu }}_{i}^{0}\left(t,L\right) & = & 0, \end{array}$$

(9) $$\begin{array}{ccc} {\bar{\rho }}_{i}^{0}\left(0,x\right) & = & {\left.{\bar{\rho }}_{i}\right|}_{U=0} \end{array}$$

where Equations (7) and (8) correspond to the open and closed boundaries, respectively; Equation (9) is the initial condition accounting for the confined electrolyte properties at U=0. Such partial differential Equation (6) with (7)–(9) can be solved numerically using c-DFT as discussed in Appendix C.

Notice, that here and below we use $\rho_{i}\equiv \rho_{i}^{0}$, $\mu \equiv \mu^{0}$ and $\psi \equiv \psi^{0}$ the dominant terms in the expansions (4) and (5) over small parameter H/L. The chemical potentials $\mu_{i}$ are defined by the popular confined fluid c-DFT model, see Appendix D. It allows us to account for the realistic behavior of the electrolytes in confinement: alteration of over-screening/crowding interfaces [43]; the capacity oscillations in narrow nanopores [6]. Such subnanoscale properties are beyond the scope of the PNP equations [24], linear TLM models [23] or their very recent modifications [17,19]. Thus, since the derived transport equation correctly describes the ions-packing properties and the electrostatic fluctuations, we can extend the theoretical study to the ultra-narrow pores comparable to molecular size.

## 3. Results

Similar to some molecular simulations [25,26], we assume that electrolyte is a symmetric two component mixture consisting of the molecules with the diameters $d_{1}=d_{2}=d$ and the charge valences $Z_{1}=-Z_{2}=1$. For the sake of simplicity, we set the diffusion coefficients of the components to be equal $D_{1}=D_{2}=D$. In the case of this symmetric electrolyte, it is also useful to introduce, following [9], the dimensionless variables $H^{*}=H/d$, $U^{*}=eU/k_{B}T$, $Q^{*}=Qd^{2}/e$, $\rho^{*}=\rho d^{3}$. It can be shown from dimensional arguments that the characteristic time for the considered problem is $L^{2}/D$ and one can also introduce the scaled time $t^{*}=tD/L^{2}=t/\tau$.

**Figure 2:**
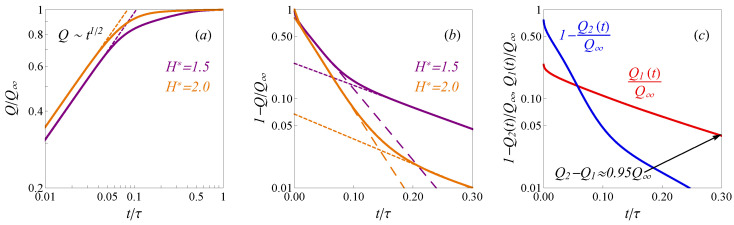
(**a**) The non-dimensional total charge (solid lines) profiles in the pores with different widths $H^{*}=1.5$ and $H^{*}=2.0$. The dashed lines indicate the square-root trends at early times. (**b**) The slit pore charging (solid lines) in the linear-log scale for $H^{*}=1.5$ and $H^{*}=2.0$. Long-dashed and short-dashed lines show two exponential trends (long-dashed and short-dashed lines) at intermediate and late times. (**c**) The separated total charge contributions from co-ions (red) counter-ions (blue) as function of time. In all plots $U^{*}=10$ and $\tau =L^{2}/D$.

The charges of electrolyte components are conveniently defined in terms of average densities as $Q_{i}\left(t\right)=-eZ_{i}HL^{-1}\int_{0}^{L}dx\;{\bar{\rho }}_{i}\left(t,x\right)$. An example of the calculated time-dependent charges Q(t) for subnanopore electrodes ($H^{*}=1.5$ and $H^{*}=2$) at sufficiently high potential $U^{*}=10$ is shown in Figure 2a,b. A discussion of the used numerical methods can be found in Appendix C. As one can see from Figure 2a, the root-square law $Q∼\sqrt{t}$ describes the notable part of the charging process at early times well. However, when the charge *Q* approaches the saturated value $Q_{\infty }$, the trend changes to the exponential one. Figure 2b shows that in the case of larger pores with $H^{*}=2$, the charging up to almost 95% is described by the following equation:

(10) $$\frac{Q}{Q_{\infty }}=1-\frac{8}{\pi^{2}}e^{-t/\tau_{1}}.$$

Expression (10) is the leading term of the analytical solution of the TLM [24]. Despite that the pores sizes H∼d and potentials $U>k_{B}T/e$ are significantly beyond the ranges of TLM applicability, the published computer simulations [25,44] show the adequacy of exponential trend in Equation (10) for fitting of the charge dependency on time. We observed that at late times the calculated profiles Q(t) in Figure 2b follow another exponential trend succeeding (10), which is notably slower and describes the charging until almost full saturation. The experimentally observed charging time for supercapacitors is around 10^3^ s, while the published dynamic DFT models result in enormously underestimated values ∼10^−9^ s [19]. The proposed model provides both the realistic behavior of the capacitance’s properties (Appendix D) and correct charging time scales. To provide numerical estimations we have used experimental parameters from refs. [19,45]: the pore length is *L* = 0.5 mm and confined diffusion coefficient *D* = 2 × 10 ^−10^ m^2^s^−1^. The full charging times estimations in the physical units for the pores $H^{*}=1.5$ and $H^{*}=2$ shown in Figure 2b correspond to around 700 s and 400 s, respectively, which agrees with the experimental characteristics.

**Figure 3:**
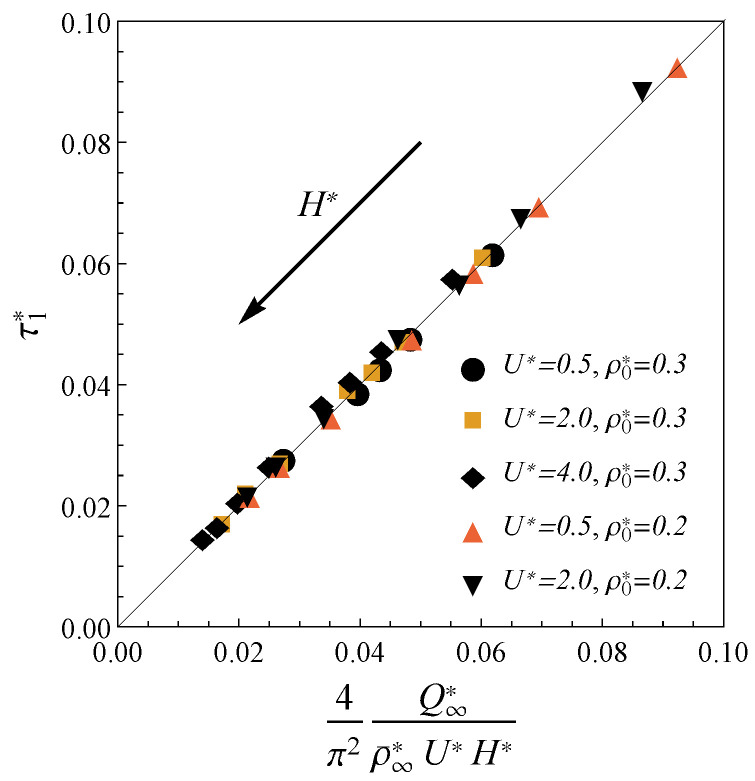
The calculated dimensionless charging time $\tau_{1}^{*}$ for the electrodes with $H^{*}>2$ at the external potentials $U^{*}\leq 4$ and the bulk electrolyte densities $\rho_{0}^{*}=\left\{0.2,0.3\right\}$ versus the scaling law (11). The arrow shows direction of the pores size increase.

Calculations for a wide range of system parameters show that the full charging (say 95% for the sake of concreteness) in pores with $H^{*}\geq 2$ is described by the first exponential trend in Equation (10). For this reason, we use Equation (10) to fit the charging time $\tau_{1}^{*}$ from the calculated profiles Q(t). Figure 3 demonstrates that the charging time for the electrodes of width $2\leq H^{*}\leq 4$ and various electrolyte bulk densities $\rho_{0}$ and applied potentials $U^{*}\leq 4$ can be explicitly expressed in terms of macroscopic parameters as

(11) $$\tau_{1}^{*}=\frac{4}{\pi^{2}}\frac{Q_{\infty }^{*}}{{\bar{\rho }}_{\infty }^{*}U^{*}H^{*}}.$$

Here, ${\bar{\rho }}_{\infty }^{*}={\bar{\rho }}_{1,\infty }^{*}+{\bar{\rho }}_{2,\infty }^{*}$ is the total final density; the coefficient $4/\pi^{2}$ is obtained from the comparison with the TLM. The potentials considered here (up to 0.1 V in dimensional terms) correspond to the lower range of the values used in the modern experiments [45,46,47], but are significantly beyond the formal applicability range of the TLM [24]. To compare result Equation (11) with TLM relaxation time we consider the limit of large pores ($H^{*}\gg 1$) at extremely low potentials ($U^{*}\ll 1$). Unlike nano-confinement, in this limit, the total density is almost constant $\bar{\rho }≃2\rho_{0}$, and the charge can be calculated from the linearization of the Gouy–Chapman theory $Q^{*}≃4\rho_{0}^{*}U^{*}\lambda_{D}/d$, where $\lambda_{D}$ is the Debye length. Inserting these approximations in Equation (11), we obtain $\tau_{\mathrm{TLM}}^{*}=4\lambda_{D}/\left(\pi^{2}h_{p}\right)$, where $h_{p}$ is the ratio of pore cross-sectional area to perimeter. Expression $\tau_{\mathrm{TLM}}^{*}$ fits the TLM relaxation time from ref. [24]. Significantly, such assumptions are not relevant to nanopores charging due to the properties of the confined electrolyte. For example, the total charge *Q* becomes an oscillating function of *H*, average densities strongly depend on the ion sizes, and electrostatic correlations play a crucial role. Thus, our result Equation (11) generalizes the TLM predictions for the case of the charging in nanopores at moderate potentials. Thus, our approach is more general than standard TLM since it describes the charging at realistic nanopores conditions.

In addition, we have not met the derived expression (11) in the literature. However, we observed an interesting connection between expression (11) and the very recent result for the charging time published in ref. [44]. The authors of ref. [44] used the effective circuit to obtain RC-time of the charging inside nanoporous Metal Organic Frameworks electrodes. The application of this adopted TLM approach from ref. [44] to slit pores gives the following expression for relaxation time

(12) $$\tau_{\mathrm{aTLM}}=\frac{4}{\pi^{2}}\frac{C_{a}L^{2}}{\sigma l},$$

where using the notation adopted in ref. [44] the areal capacitance can be expressed as $C_{a}=Q_{\infty }/2U$; the ionic conductivity inside the pore can be expressed in terms of the electrical mobility of ions $m=eD/k_{B}T$ and ions density ${\bar{\rho }}_{\infty }$ as $\sigma =e{\bar{\rho }}_{\infty }m$. Inserting these electrolyte characteristics into Equation (A51) one can obtain that $\tau_{\mathrm{aTLM}}^{*}=\tau_{1}^{*}$, see details in Appendix E. Therefore, Equation (11) is equivalent to one from the nanopores RC-circuit developed in ref. [44]. The advantage of our approach over the adopted TLM is self contained form provided by c-DFT’s calculations without highly cost simulations.

The three consecutive dynamic regimes predicted in our work describe the results of molecular dynamics simulations published in refs. [25,26,27]. Similarly with ref. [25,26] we observed a stark contrast between the charging dynamics inside ultra-narrow $H^{*}=1.5$ and wider $H^{*}=2$ pores shown in Figure 2, for comparison, see Figure 2 and Figure 3 in ref. [26] and ref. [25], respectively. In Figure 2b the first exponential regime (10) for $H^{*}=1.5$ covers the charging up to only 75%. Therefore, the significant part of the full charging inside ultra-narrow pores ($H^{*}=1.5$) is defined by the slower second exponential trend: the charge to 95% is ∼2 times slower than in the case of wider pores $H^{*}=2$.

**Figure 4:**
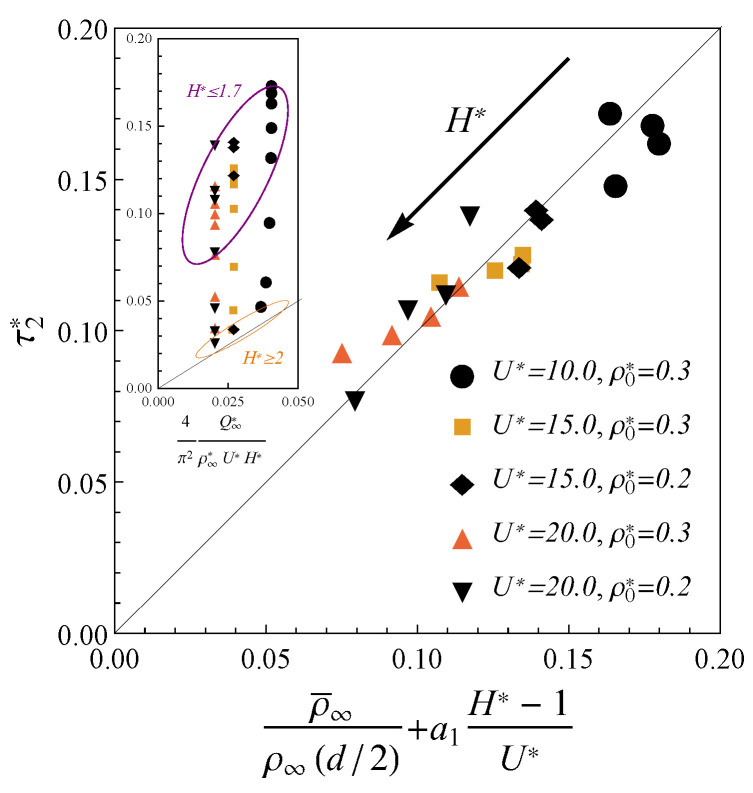
The dimensionless charging timescale $\tau_{2}^{*}$ for the electrodes with $1.3\leq H^{*}\leq 1.7$ at the high external potentials $10\leq U^{*}\leq 20$ versus the scaling law (13). The inset shows the same timescale versus the scaling law (11); the pores with $H^{*}\leq 1.7$ and $H^{*}\geq 2.0$ are marked with purple and orange ellipses.

To investigate this effect numerically, we described the charge profiles near the saturation $Q\left(t\right)∼0.95Q_{\infty }$ in terms of another exponential trend $Q/Q_{\infty }=1-A_{2}e^{-t/\tau_{2}}$, where $\tau_{2}\geq \tau_{1}$ and $A_{2}$ are the fitting parameters. The results for the corresponding relaxation time $\tau_{2}^{*}$ are shown in the inset of Figure 4. As one can see from this inset, expression (11) fits the charging time in larger pores ($H^{*}\geq 2$). However, we observed that in the case of the ultra-narrow pores $H^{*}\leq 1.7$, charging slow down becomes notable. We observed that the charging slowdown starts as the pore width becomes less than two molecular diameters ($H^{*}<2$). In such confinement, the electrolyte behavior near the wall crucially influence on the ions distribution inside whole pore. Accordingly, it is reasonable to correlate the charging inside narrow pores not only with the average inner density $\bar{\rho }$ but the wall-contact density as well. As one can see from Figure 4, the charging time in ultra-narrow pores ($H^{*}\leq 1.7$) at high potential can be fitted to the following expression

(13) $$\tau_{2}^{*}=\frac{{\bar{\rho }}_{\infty }}{\rho_{\infty }\left(d/2\right)}+a_{1}\frac{H^{*}-1}{U^{*}}$$

where $\rho_{\infty }\left(d/2\right)=\rho_{\infty ,1}\left(d/2\right)+\rho_{\infty ,2}\left(d/2\right)$ is the wall-contact density at the final state of complete charging and $a_{1}≃0.6$ is the fitting parameter. The inverse dependence of charging time (13) on the potential *U* contributes as the pore’s width increases. This behavior is consistent with the high potential limit ($H^{*}{\bar{\rho }}_{\infty }^{*}≃Q_{\infty }^{*}$) of expression (11), which fits the data well around $H^{*}=2$.

To discuss the slowdown dynamics inside ultra-narrow pores we considered the dynamics of the co-ions/counter-ions separately. We observed that the contributions to total charge from the co-ions $Q_{1}\left(t\right)$ and counter-ions $Q_{2}\left(t\right)$ shown in Figure 2c exhibit significantly different relaxation times. More precisely, the counter-ions adsorption proceed more rapidly than slow outflux of the co-ions. Therefore, at the late times the counter-ions contribution is almost equal to the final charge $Q_{2}≃Q_{\infty }$, while the co-ions’ slow-release decrease the total charge $Q_{\infty }-Q_{1}$. The possible explanation of this phenomenon is that the co-ions are trapped in the crowded counter-ion phase. Such behavior of the ions in nanopores was observed in MD simulations of symmetric electrolyte [26,27]. To demonstrate that expression (13) is in agreement with this explanation, we consider the ultra-narrow pores H∼d. Then, the second term in Equation (13) is negligible and the counter-ion density dominates ${\bar{\rho }}_{\infty ,2}\gg {\bar{\rho }}_{\infty ,1}$. Therefore, the relaxation time (13) can be written as $\tau_{2}=L^{2}{\bar{\rho }}_{\infty ,2}/D\rho_{\infty ,2}\left(d/2\right)$. At the late times, such dynamics is equivalent to a diffusion process of co-ions with the effective diffusion coefficient $D_{1}={\bar{\rho }}_{\infty ,2}D/\rho_{\infty ,2}\left(d/2\right)$, while the counter ions component is already in equilibrium. This coefficient $D_{1}<D$ is defined by the density and structure of the counter-ion component distribution, that confirms the idea that the the abrupt counter-ions adsorption induces slow diffusion of the co-ions.

This paper proved the concept of H/L-expansion in application to the nanoporous charging compared with the experiment and simulations results. This approach could be helpful in applications beyond the charging time calculations. For example, we recently became aware of paper [48], where the authors use the same asymptotic approach to calculate the impedance response in slit pores. In the future, the developed model could be perspective in the following research directions.

(I): We considered only the popular version of the Constant Potential Method (CPM)—the charging starts when the surface potential suddenly steps from zero to some value. However, the CPM mode does not cover charging at a constant electric current widely used in Galvanostatic Charge–Discharge (GCD) measurements. Additionally, recent MD simulations in ref. [27] show that the charging in nanopores can be accelerated applying time-dependent potential U(t), which allows one to avoid ion clogging near the orifice. First of all, to describe the GCD- and U(t)-modes for the realistic supercapacitor’s setup, one needs to account for the dynamics of the neutral electrolyte stored in the finite bulk volume between two oppositely charged porous electrodes. Therefore, the proposed model should be improved by accounting for the bulk electrolyte, as recently done for the TLM in ref. [17], and for PNP equations in ref. [49]. Then, the time-dependent potential can be accounted for in boundary condition (8), assuming that U(t) varies slowly then the diffusion time scale in *z*-direction $H^{2}/D$. Possible realization of GCD mode follows from very recent ref. [50]—one can use the boundary condition at x=0 which indicates an applied current with the given period and amplitude (see Equation (7c) in [50]). (II): Our model is applied to the porous electrodes with flat smooth inner surface. Recently we developed model for the electrolytes structure near the electrodes with rough surfaces ref. [51]. Therefore, the proposed approach could be extended to the porous materials with rough surfaces accounting for the modified Poisson equation from ref. [51]. (III): We considered purely electrostatic supercapacitors, also known as electric double-layer capacitors. However, our approach can be extended to the pseudocapacitors’ charging mechanism based on the Faradaic processes (redox reactions). For this case, the possible modifications are discussed in ref. [21], where the authors extended the porous electrode theory from [20], accounting for the Faradaic reaction rate. Notice that these fast reactions differ from the charging mechanism of Li-ion batteries, where the chemical reactions induce the changes in the electrode molecules making/breaking chemical bonds.

## 4. Conclusions

In conclusion, we developed a model of the charging dynamics in slit nanopores accounting for the confined properties of the electrolyte. We made significant simplification of the dynamic equations exploiting the slit-pore geometry but remaining the properties of the confined electrolytes. The developed model demands much lower computational resources than computer simulations and allows the investigation of a wide range of electrolyte and electrode parameters. Unlike the previous c-DFT applications to the ion dynamics, our results for the charging time coincide with experimental data well [19]. In addition, we described three consequent regimes of the supercapacitor’s charging: the initial root-square diffusion and two exponential regimes with notably different time scales. These results agree with molecular dynamic simulations published in refs. [25,26,27]. We identified a threshold pore width below which the second exponential regime defines the full charging time. In this case, the charging inside ultra-narrow pores is notably slower and depends on the contact wall electrolytes density. These numerical estimations are crucial to avoid power density decrease using the ultra-narrow pores. Thus, the developed model will help investigate the relations between the supercapacitors’ storage and dynamics properties.

## Figures and Tables

**Figure A1:**
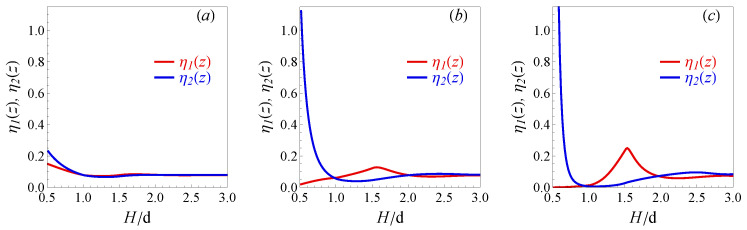
The dimensionless density distributions of positive $\eta_{1}\left(z\right)=v\rho_{1}\left(z\right)$ and negative $\eta_{2}\left(z\right)=v\rho_{2}\left(z\right)$ electrolyte’s components inside pore $H^{*}=6$ at various applied potentials $U^{*}=0.5$ (**a**), $U^{*}=5$ (**b**) and $U^{*}=20$ (**c**). These calculations correspond to the bulk density $\rho_{0,1}^{*}=\rho_{0,2}^{*}=0.15$ at the temperature $T^{*}=0.15$.

## Data Availability

Not applicable.

## Abbreviations

The following abbreviations are used in this manuscript:

c-DFT	Classical density functional theory
TLM	Transmission line model
MD	Molecular dynamics

## Appendix A. Derivation of Pore Cross-Section Averaged Equations

Here we the describe derivation of the pore cross-section averaged equations

(A1) $$\partial_{t}{\bar{\rho }}_{i}-\beta D_{i}\partial_{x}\left({\bar{\rho }}_{i}\partial_{x}{\bar{\mu }}_{i}\right)=0$$

from the original three dimensional system

(A2) $$\partial_{t}\rho_{i}-\beta D_{i}\nabla \left(\rho_{i}\nabla \mu_{i}\right)=0,$$

(A3) $$\mu_{i}=k_{B}T\log\left(\rho_{i}\Lambda^{3}\right)+eZ_{i}\psi +\frac{\delta f_{\mathrm{exc}}}{\delta \rho_{i}},$$

(A4) $$\beta e\Delta \psi =-4\pi l_{B}\sum_{i=1}^{n}Z_{i}\rho_{i}.$$

Please refer to the main document for context and notation.

We consider transport along the slit pore aligned with the *x*-axis and perpendicular to *z*-axis. The aspect ratio of the pore is assumed to be large, and accordingly, we adopt classical thin-film / lubrication approximation scaling. The pore width *H*, length *L*, and applied voltage *U* are used as scales for *z* coordinate, *x* coordinate, and potentials $\mu_{i}$, respectively. Additionally, for the sake of concreteness we scale $D_{i}$ for different components with the value of the first component diffusion coefficient *D* at bulk conditions. Timescale of the problem is defined by $L^{2}/D\beta eU$ and characteristic density is $\beta eU/4\pi l_{B}H^{2}$.

After scaling, the dimensionless transport and Poisson equations are

(A5) $$\delta \partial_{t}\rho_{i}-\delta \partial_{x}\left(K_{i}\partial_{x}\mu_{i}\right)-\partial_{z}\left(K_{i}\partial_{z}\mu_{i}\right)=0,$$

(A6) $$\delta \partial_{xx}\psi +\partial_{zz}\psi -\sum_{i=1}^{n}Z_{i}\rho_{i}=0.$$

Here, $\delta =H^{2}/L^{2}\ll 1$ and the additional definition $K_{i}\;=\;D_{i}\rho_{i}$ is introduced.

The system is supplied with boundary conditions enforcing zero fluxes

(A7) $$-K_{i}\partial_{z}\mu_{i}=0,\;z=0,1.$$

and the value of electrostatic potential

(A8) ψ=1,z=0,1.

at the channel walls.

We seek a formal asymptotic expansion of the densities $\rho_{i}$ and the potential ψ in power series of δ→0

$$\begin{array}{ccc} \rho_{i} & = & \rho_{i}^{0}+\delta \rho_{i}^{1}+\cdots ,\\ \psi & = & \psi^{0}+\delta \psi^{1}+\cdots . \end{array}$$

Substituting the latter expansion to (A5)–(A8) and collecting terms of the same order, one can obtain the *O*(1) *problem*:

(A9) $$-\partial_{z}\left(K_{i}^{0}\partial_{z}\mu_{i}^{0}\right)=0,$$

(A10) $$\partial_{zz}\psi^{0}-\sum_{i=1}^{n}Z_{i}\rho_{i}^{0}=0,$$

(A11) $$-K_{i}^{0}\partial_{z}\mu_{i}^{0}=0,\;z=0,1.$$

(A12) $$\psi^{0}=1,\;z=0,1.$$

Here, $K_{i}^{0}=D_{i}\rho_{i}^{0}$, $\mu_{i}^{0}=\mu_{i}\left[\rho_{1}^{0},...,\rho_{n}^{0},\psi^{0}\right]$. Solution of the $O\left(1\right)$ problem will be discussed below. Here we only note that using (A9) and the boundary condition (A11), one can also promptly get

(A13) $$\partial_{z}\mu_{i}^{0}=0.$$

Details of O(1) problem are further considered in Appendix B.

$O\left(\delta \right)$*problem*:

(A14) $$\partial_{t}\rho_{i}^{0}-\partial_{x}\left(K_{i}^{0}\partial_{x}\mu_{i}^{0}\right)-\partial_{z}\left(K_{i}^{0}\partial_{z}\mu_{i}^{1}+K_{i}^{1}\partial_{z}\mu_{i}^{0}\right)=0,$$

(A15) $$\partial_{xx}\psi^{0}+\partial_{zz}\psi^{1}-\sum_{i=1}^{n}Z_{i}\rho_{i}^{1}=0,$$

(A16) $$-K_{i}^{0}\partial_{z}\mu_{i}^{1}-K_{i}^{1}\partial_{z}\mu_{i}^{0}=0,\;z=0,1.$$

(A17) $$\psi^{1}=0,\;z=0,1.$$

Here $K_{i}^{1}\;=\;D_{i}\rho_{i}^{1}$ and

$$\mu_{i}^{1}\;=\;\sum_{j=1}^{n}\frac{\delta \mu_{i}}{\delta \rho_{j}}\left[\rho_{1}^{0},...,\rho_{n}^{0},\psi^{0}\right]\rho_{j}^{1}+\frac{\delta \mu_{i}}{\delta \psi }\left[\rho_{1}^{0},...,\rho_{n}^{0},\psi^{0}\right]\psi^{1}.$$

The solution of the $O\left(\delta \right)$ problem is beyond the scope of the study. We limit ourselves to consideration of dynamical problem in the leading order of approximation and use $O\left(\delta \right)$ problem for rigorous derivation of the averaged equations only.

Integrating (A14) over the pore width and using boundary conditions (A16) and corollary (A13) one can get

(A18) $$\partial_{t}{\bar{\rho }}_{i}^{0}-\partial_{x}\left({\bar{K}}_{i}^{0}\partial_{x}{\bar{\mu }}_{i}^{0}\right)=0.$$

Here, $\bar{f}=\int_{0}^{1}dzf$. The condition (A13) is used here while integrating the second term of (A14) by parts and to replace $\mu_{i}^{0}$ by ${\bar{\mu }}_{i}^{0}$. The Equation (6) is essentially (A18) written in dimensional terms after dropping superscripts.

## Appendix B. *O*(1) Problem

Casting the Equations (A10), (A12) and (A13) back to dimensional variables we get

(A19) $$\beta e\partial_{zz}\psi^{0}=-4\pi l_{B}\sum_{i=1}^{n}Z_{i}\rho_{i}^{0},$$

(A20) $$\psi^{0}=U,\;z=0,H,$$

(A21) $$\partial_{z}\mu_{i}^{0}=0.$$

The solution of (A19) with boundary conditions (A20) can be written in the integral form

(A22) $$\beta e\psi^{0}=\beta eU+\frac{4\pi l_{B}z}{H}\int_{0}^{H}dz^{\prime }\left(H-z^{\prime }\right)\sum_{i=1}^{n}Z_{i}\rho_{i}^{0}-4\pi l_{B}\int_{0}^{z}dz^{\prime }\left(z-z^{\prime }\right)\sum_{i=1}^{n}Z_{i}\rho_{i}^{0}.$$

It follows from (A21) that $\mu_{i}^{0}$ does not depend on the *z* coordinate $\mu_{i}^{0}={\bar{\mu }}_{i}^{0}\left(t,x\right)$. Substituting the latter to (2) and rearranging terms, one can get the following equation for the density distribution across the pore

(A23) $$\rho_{i}^{0}=\frac{1}{\Lambda^{3}}\exp\left(\beta {\bar{\mu }}_{i}^{0}\right)E_{i}^{0}.$$

Here,

$$E_{i}^{0}=\exp\left(-\beta eZ_{i}\psi^{0}-\beta \frac{\delta f_{\mathrm{exc}}}{\delta \rho_{i}}\left[\rho_{1}^{0},...,\rho_{n}^{0}\right]\right)$$

and further details on $f_{\mathrm{exc}}$ can be found in Appendix D.

Formal integration of (A23) over z∈[0,H] and the fact that ${\bar{\mu }}_{i}^{0}={\bar{\mu }}_{i}^{0}\left(t,x\right)$ allow to eliminate the chemical potential ${\bar{\mu }}_{i}^{0}$ and rewrite the equation in terms of average density ${\bar{\rho }}_{i}^{0}$ as

(A24) $$\rho_{i}^{0}={\bar{\rho }}_{i}^{0}\frac{E_{i}^{0}}{{\bar{E}}_{i}^{0}}.$$

We look for effectively 1D equilibrium densities’ distributions depending on *t* and *x* only parametrically via the average densities $\rho_{i}^{0}=\rho_{i}^{0}\left({\bar{\rho }}_{i}^{0}\left(t,x\right),z\right)$. Once the distributions are found from (A24) and (A22), one can evaluate (2) at any coordinate *z*, and thus, get the potentials as functions of average densities ${\bar{\mu }}_{i}^{0}={\bar{\mu }}_{i}^{0}\left({\bar{\rho }}_{i}^{0},...,{\bar{\rho }}_{n}^{0}\right)$.

## Appendix C. Notes on Numerical Solution

Numerical solution of the transport Equation (6) involves two different tasks: solution of the dynamic equations given the potentials as function of densities and evaluation of potentials themselves.

For a given potential, spatial discretization of the system (6) is performed on a uniform staggered grid using finite volume method. The resulting system of nonlinear ODEs is solved by the built-in method of Wolfram Mathematica [52].

The potentials as functions of densities are defined by the solution of the $O\left(1\right)$ problem described in Appendix B. The system of Equation (A24) for i=1,⋯,n comprises the fixed-point problem with the right-hand side defined by the dependency of excess energy variation on densities described in Appendix D and the solution (A22) of the Poisson Equations (A19) and (A20). Given ${\bar{\rho }}_{i}^{0}$, it is solved by the classical Picard iterations with underrelaxation involving intermediate step of (A19) and (A20) solution for current densities guess.

For the sake of computational efficiency, the potentials ${\bar{\mu }}_{i}^{0}$ are not evaluated “on the fly" during the solution of the dynamic Equation (6). Instead, the potentials are first calculated on a sufficiently fine grid in average densities space. Next, smooth interpolation is built based on the calculated values. Then, the interpolants are used while solving the dynamic problem.

## Appendix D. Density Functional Theory

Here, we describe in detail the thermodynamic model of an electrolyte inside a nanopore, which is based on classical density functional theory (c-DFT). The version of this approach developed for neutral molecules is able to take into account the influence of nanoscale geometrical constraints [41]. The confined fluid model can be extended to electrolyte fluids accounting for electrostatic correlations and external Coulomb field [42]. We consider an open slit pore stored by neutral electrolyte mixture $\sum_{k=1}^{n}Z_{k}\rho_{k,b}=0$ with known composition and chemical potentials ${\left\{\mu \right\}}_{k=1}^{n}$. Such confined system is described in terms of the Grand Canonical potential Ω and external field:

(A25) $$\Omega \left[\left\{\rho_{i}\left(r\right)\right\}\right]=F\left[\left\{\rho_{i}\left(r\right)\right\}\right]+\sum_{i=1}^{n}\int dr\rho_{i}\left(r\right)\left(U_{\mathrm{ext},i}\left(r\right)-\mu_{i}\right),$$

where $U_{\mathrm{ext}}$ is the external field acting on a fluid molecule, μ is the chemical potential. In the case of charged molecules, the external fields contains not only wall potential, but also Coulomb field contribution:

(A26) $$U_{\mathrm{ext},i}=U_{w,i}+U_{C,i}.$$

In our study, we use the hard sphere potential to described non-electrostatic fluid-solid interactions:

(A27) $$U_{w,i}\left(r\right)=\{\begin{array}{ccc} \infty & \mathrm{if} & r<d_{i}/2\\ 0 & \mathrm{if} & r>d_{i}/2 \end{array}.$$

The slit pore geometry allows us to reduce the spatial density distribution $\rho_{i}\left(r\right)$ to the 1D function $\rho_{i}\left(z\right)$ of the normal distance to the solid surface. The Helmholtz energy of ionic liquids can be written as follows:

(A28) $$F=F_{\mathrm{id}}+F_{\mathrm{hs}}+F_{C}+F_{\mathrm{el}},$$

where $F_{\mathrm{id}}$ is the ideal gas contribution; $F_{\mathrm{hs}}$ is the hard sphere term accounting for ionic excluded volume effects; $F_{C}$ is the Coulomb interaction; $F_{\mathrm{el}}$ is the residual electric contribution. Here, only the ideal part is known exactly:

(A29) $$F_{\mathrm{id}}=Ak_{B}T\sum_{i=1}^{n}\int dz\rho_{i}\left(z\right)\log\left(\left[\Lambda^{3}\rho \left(z\right)\right]-1\right).$$

The remaining terms define the excess part of the total Helmholtz free energy:

(A30) $$F_{\mathrm{exc}}=F_{\mathrm{hs}}+F_{C}+F_{\mathrm{el}}$$

In accordance with the DFT approach, the equilibrium density distributions are defined by the following system:

(A31) $$\frac{\delta \Omega }{\delta \rho_{i}}=0,\;i=1,...,n$$

After substitution of expressions for the Helmholtz free energy (A28) and the external potential (A26), the conditions (A31) take a more explicit form:

(A32) $$\rho_{i}=\rho_{i}^{0}\exp\left[-\beta U_{C,i}-\beta U_{w,i}-\lambda_{i}\right],$$

where $\rho_{i}^{0}$ is the bulk component density, $\lambda_{i}$ is the density derivative of the deviation of the excess terms from the bulk ones:

(A33) $$\lambda_{i}=\beta \frac{\delta (F_{\mathrm{exc}}-F_{\mathrm{exc}}^{0})}{\delta \rho_{i}}$$

where $F_{\mathrm{exc}}^{0}$ is the bulk excess free energy corresponding to the homogeneous mixture ${\left\{\rho_{k}^{0}\right\}}_{k=1}^{n}$.

The functional derivative of the Coulomb contribution has the following form:

(A34) $$\beta \frac{\delta F_{C}}{\delta \rho_{i}}=Z_{i}l_{B}\sum_{j=1}^{n}Z_{j}\int ds\frac{\rho_{j}\left(s\right)}{\left|s-r\right|},$$

where $l_{B}=\beta e^{2}/4\pi \epsilon \epsilon_{0}$ is the Bjerrum length. The right hand of expression (A34) can be rewritten in terms of external Coulomb field $U_{C}$ and the mean electrostatic potential ψ:

(A35) $$Z_{i}e\psi -U_{C,i}=Z_{i}l_{B}\sum_{j=1}^{n}Z_{j}\int ds\frac{\rho_{j}\left(s\right)}{\left|s-r\right|}$$

Using expressions (A34) and (A35) Equation (A32) can be rewritten as follows:

(A36) $$\rho_{i}=\rho_{i}^{\left(0\right)}\exp\left[-\beta U_{w,i}-\beta Z_{i}e\psi -\frac{\beta \delta }{A\delta \rho }\left(\Delta F_{\mathrm{hs}}+\Delta F_{\mathrm{el}}\right)\right]$$

where symbol Δ means the difference between confined and bulk energies.

The hard sphere contribution can be calculated using Fundamental Measure Theory [41] as:

(A37) $$\beta F_{\mathrm{hs}}\left[\rho_{1}\left(z\right),...,\rho_{n}\left(z\right)\right]=A\int_{0}^{H}dz^{\prime }\Phi \left(n_{0},n_{1},n_{2},n_{3},n_{v1},n_{v2}\right)$$

where the function Φ depends on the weighted densities $n_{\alpha }$ defined as:

(A38) $$\begin{array}{cc} n_{0} & =\sum_{k=1}^{n}\frac{1}{d_{i}}\int_{z-R}^{z+R}\rho_{k}\left(z^{\prime }\right)dz^{\prime }\\ n_{1} & =\frac{1}{2}\sum_{k=1}^{n}\int_{z-R}^{z+R}\rho_{k}\left(z^{\prime }\right)dz^{\prime }\\ n_{2} & =\sum_{k=1}^{n}\pi d_{i}\int_{z-R}^{z+R}\rho_{k}\left(z^{\prime }\right)dz^{\prime }\\ n_{3} & =\frac{1}{2}\sum_{k=1}^{n}\int_{z-R}^{z+R}\left[d_{k}^{2}-{\left(z-z^{\prime }\right)}^{2}\right]\rho_{k}\left(z^{\prime }\right)dz^{\prime }\\ n_{v1} & =-\sum_{k=1}^{n}\frac{1}{d_{k}}\frac{z}{z}\int_{z-R}^{z+R}dz^{\prime }\left(z^{\prime }-z\right)\rho \left(z^{\prime }\right)\\ n_{v2} & =-2\pi \sum_{k=1}^{n}\frac{z}{z}\int_{z-R}^{z+R}dz^{\prime }\left(z^{\prime }-z\right)\rho \left(z^{\prime }\right) \end{array}$$

We use one of the most popular version [9] of the Φ defined as:

(A39) $$\Phi =-n_{0}\log\left(1-n_{3}\right)+\left(n_{1}n_{2}-n_{v1}n_{v2}\right)/\left(1-n_{3}\right)+1/\left(36\pi \right)\left(n_{3}\log\left(1-n_{3}\right)+n_{3}^{2}/{\left(1-n_{3}\right)}^{2}\right)\left(n_{2}^{3}-3n_{2}n_{v2}^{2}\right)/n_{3}^{3}$$

Therefore, the functional derivative of the hard sphere contribution can be calculated by the following way:

(A40) $$\frac{\beta \delta F_{\mathrm{hs}}}{\delta_{i}\rho \left(z\right)}=A\int_{0}^{H}dz^{\prime }\sum_{\alpha }\frac{\partial \Phi (n_{\alpha })}{\partial n_{\alpha }}\frac{\delta n_{\alpha }}{\delta \rho_{i}}$$

To calculate electrostatic term in Equation (A36), we use the approach described in ref. [42]:

(A41) $$\frac{\delta \Delta F_{\mathrm{el}}}{\delta \rho_{i}}=-\sum_{k=1}^{n}\int ds{\bar{c}}_{ki}\left(r,s\right)\Delta \rho_{k}\left(s\right)$$

where the weighted correlation function defined as

(A42) $${\bar{c}}_{ki}\left(r,s\right)=\frac{\int c_{ki}\left(r^{\prime },s\right)f_{ki}\left(r^{\prime }\right)}{\int dr^{\prime }f_{ki}\left(r^{\prime }\right)}$$

In our work, we use the FMT/WCA-k^2^ approach, which corresponds to the following expression of *f*-function:

(A43) $$f_{ki}\left(r^{\prime }\right)=\kappa^{2}\left(r^{\prime }\right)\Theta (|r-r^{\prime }\left|-d_{ki}\right)$$

where $d_{ki}=\left(d_{k}+d_{i}\right)/2$ is the average diameter, and κ is the inverse Debye parameter given by

(A44) $$\kappa^{2}\left(r^{\prime }\right)=4\pi l_{B}\sum_{k}^{n}\rho_{k}Z_{k}$$

In accordance with work [42], here we use the approximated analytical expression for the weighted correlation function in terms of MSA solution:

(A45) $${\bar{c}}_{ki}\left(r^{\prime },s\right)≃c_{ki}^{MSA}\left(\right|r^{\prime }-s\left|\right)=\beta U_{ki}\left(r\right){\left[1-B_{ki}\left(r^{\prime }\right)\frac{r}{d_{ki}}\right]}^{2}\Theta \left(d_{ki}-r\right)$$

where $r=|r^{\prime }-s|$ is the scalar distance, $B_{ki}$ depends analytically on Debye parameter κ (A44) as

(A46) $$B_{ki}=\frac{1+\kappa d_{ki}-\sqrt{1+2\kappa d_{ki}}}{\kappa d_{ki}}$$

Therefore, the weighted correlation function (A42) in slit geometry ρ(z) can be written as:

(A47) $${\bar{c}}_{ki}\left(r\right)=\beta U_{ki}\left(r\right)\left[1-2B_{1,ki}\left(z\right)\frac{r}{d_{ki}}+B_{2,ki}\left(\frac{r}{d_{ki}}\right)\right]\Theta \left(d_{ij}-r\right)$$

where

(A48) $$B_{m,ki}\left(z\right)=\frac{\int_{z-d_{ki}}^{z+d_{ki}}dz^{\prime }B^{m}\left(z^{\prime }\right)\kappa^{2}\left(z^{\prime }\right)\left(d^{2}-{\left(z-z^{\prime }\right)}^{2}\right)}{\int_{z-d_{ki}}^{z+d_{ki}}dz^{\prime }\kappa^{2}\left(z^{\prime }\right)\left(d^{2}-{\left(z-z^{\prime }\right)}^{2}\right)}$$

In our work, we use the same system parameters as in ref. [9] corresponding to the dimensionless temperature $T^{*}=d/l_{B}=0.15$. The characteristic dependence of the density distribution profiles on applied potential is shown in Figure A1. As shown in [9], such behavior of co- and counter-ions results in the oscillating capacity properties.

## Appendix E. Connection with Transition Line Model (TLM)

Here we supplement the analysis of the first exponential regime’s time scale

(A49) $$\tau_{1}^{*}=\frac{4}{\pi^{2}}\frac{Q_{\infty }^{*}}{{\bar{\rho }}_{\infty }^{*}U^{*}H^{*}}$$

by another connection with the result of TLM. In ref. [44] the TLM solution was used to describe the charging inside nanoporous metal organic frameworks (MOFs) electrodes. The following equation for the net pore charge after jump-wise application of constant potential was obtained:

(A50) $$\frac{Q(t)}{Q_{\infty }}=1-\frac{2}{\pi^{2}}\sum_{n=0}^{\infty }\frac{\exp\left[-\pi^{2}{\left(n+1/2\right)}^{2}{\left(l/L\right)}^{2}t/\tau \right]}{{\left(n+1/2\right)}^{2}}.$$

Here $\tau =C_{a}l/\sigma$ is the intrinsic relaxation time, $C_{a}$ is the areal capacity of the pore, *l* is the pore volume divided by its surface area, σ is the ionic conductivity inside the pore. Please note that the exponent in (A50) differs from the given in the original paper by the factor of 4, because the symmetric pore connected to bulk electrolyte at both ends and effectively composed of two closed-end pores considered here was studied in [44].

The series in the right hand of expression (A50) can be approximated by the leading term n=0, that result in the following time-scale of the TLM model:

(A51) $$\tau_{\mathrm{aTLM}}=\frac{4}{\pi^{2}}\frac{C_{a}L^{2}}{\sigma l}.$$

For the slit pore geometry l≈H/2. Using the notation adopted in this paper the areal capacitance can be expressed as $C_{a}=Q_{\infty }/2U$. Finally, the ionic conductivity inside the pore can be expressed in terms of the electrical mobility of ions $m=eD/k_{B}T$ and ions density ${\bar{\rho }}_{\infty }$ as $\sigma =e{\bar{\rho }}_{\infty }m$. Substitution of these expressions to (A51) gives

(A52) $$\tau_{\mathrm{aTLM}}=\frac{4}{\pi^{2}}\frac{Q_{\infty }k_{B}T}{e^{2}{\bar{\rho }}_{\infty }UH}\frac{L^{2}}{D}.$$

Introducing dimensionless variables $H^{*}=H/d$, $U^{*}=eU/k_{B}T$, $Q^{*}=Qd^{2}/e$, $\rho^{*}=\rho d^{3}$ and $t^{*}=tD/L^{2}$ used in the main document into the latter expression one can readily obtain that $\tau_{\mathrm{aTLM}}^{*}=\tau_{1}^{*}$, i.e. TLM time scale is equivalent to the time given by (A49).
